# Assessment of subclinical cardiovascular alterations in nonfunctioning adrenal incidentalomas

**DOI:** 10.55730/1300-0144.5360

**Published:** 2022-01-16

**Authors:** Bülent CAN, Pelin KARACA ÖZER, Büşra CAN, Özge TELCİ ÇAKLILI

**Affiliations:** 1 Department of Internal Medicine, Division of Endocrinology and Metabolism, Faculty of Medicine, İstanbul Medeniyet University, İstanbul, Turkey; 2 Department of Cardiology, Faculty of Medicine, İstanbul University, İstanbul, Turkey; 3 Department of Internal Medicine, Division of Geriatrics, Faculty of Medicine, Marmara University, İstanbul, Turkey; 4 Department of Internal Medicine, Division of Endocrinology and Metabolism, Faculty of Medicine, İstanbul University, İstanbul, Turkey

**Keywords:** Adrenal incidentaloma, carotid intima-media thickness, epicardial adipose tissue thickness, left ventricular mass

## Abstract

**Background/aim:**

Adrenal incidentalomas have been associated with increased cardiovascular risk and have a prevalence as high as 10%. This study aims to evaluate carotid-intima media thickness (CIMT), left ventricular mass, and epicardial adipose tissue thickness in nonfunctioning adrenal incidentaloma patients and compare their results with healthy controls.

**Materials and methods:**

Patients who were referred to the endocrinology clinic for adrenal incidentaloma between 2014 and 2019 were assessed with 1 mg dexamethasone suppression test, 24-h urine metanephrines and normetanephrines, plasma aldosterone to renin ratio. Age and gender-matched subjects without an adrenal mass formed the control group. Left ventricular mass, epicardial adipose tissue thickness, and CIMT of both groups were measured.

**Results:**

A total of 41 adrenal incidentaloma patients (21 female, 52.5%) and 40 healthy controls (19 female, 46.3%) were included in the study. Patients with adrenal incidentalomas had increased CIMT. No differences were observed in left ventricle mass or epicardial adipose tissue thickness. There was no correlation between CIMT and adenoma size or serum cortisol (p = 0.2 and p = 0.6, respectively). There was a statistically significant correlation between CIMT and age (p = 0.016, r = 0.295). HBA1c (p = 0.001) and age (p = 0.05) were independently associated with CIMT in regression analysis.

**Conclusion:**

Adrenal incidentaloma patients need to be monitored for cardiac dysfunction. CIMT may be used to evaluate adrenal incidentaloma patients for early cardiovascular risk.

## 1. Introduction

Adrenal incidentalomas (AI) are defined as adrenal masses with a diameter >1 cm, discovered on an imaging test performed for a condition unrelated to adrenal disease [1]. The incidence of AI has increased over the years with the widespread use of imaging techniques for thoracic and abdominal regions [2]. Its prevalence is 1%–8.7% in autopsy series and as high as 10% in older adults [1].

Adrenal diseases, such as Cushing’s syndrome, primary aldosteronism, and pheochromocytoma, pose a risk for cardiovascular disease [3–5]. Moreover, cardiovascular risk has been reported to increase even if adrenal incidentalomas are nonfunctioning [6,7]. Metabolically, AI has been associated with type 2 diabetes, dyslipidaemia, and hypertension [7].

Carotid intima-media thickness (CIMT) and epicardial adipose tissue thickness (EATT) are considered to be noninvasive markers of atherosclerosis [8–10]. Left ventricular mass (LVM) is associated with increased cardiovascular risk and mortality [11]. Alterations of these parameters are considered to be predictors of early cardiovascular disease. In this study, we aimed to evaluate CIMT, LVM, and EATT in patients with AI and compare their results with healthy controls.

## 2. Subjects and methods

### 2.1. Study population and design

Patients who were referred to the endocrinology clinic for AI between 2014 and 2019 were evaluated. Adrenal lesions were discovered by computed tomography (CT) or magnetic resonance imaging (MRI), which were performed for unrelated conditions such as urolithiasis. The radiologic characteristics of all AI were consistent with benign cortical adenoma (demarcated lesions less than 4 cm with attenuation values lower than 10 Hounsfield Units). Patients who were older than 18 years and gave written consent were included in the study. Laboratory tests including 1 mg dexamethasone suppression test, 24-h urine metanephrines, and normetanephrines, plasma aldosterone to renin ratio were performed to detect any functional adrenal masses. If serum cortisol after 1 mg overnight dexamethasone suppression test was above 1.8 mcg/dL, a two-day (2 mg) dexamethasone test was administered. Patients with functioning adenomas were excluded.

Patients diagnosed with diabetes according to the criteria of the American Diabetes Association were excluded (diabetes mellitus was defined as fasting plasma glucose >126 mg/dL (7 mmol/L), oral glucose tolerance test 2h plasma glucose >200 mg/dL (11.1 mmol/L) and HbA1c ≥6.5%). Patients on antihypertensive treatment or with systolic blood pressure higher than 140 mmHg and/or diastolic blood pressure higher than 90 mmHg were also excluded. Patients with a body mass index (BMI) above 35 were excluded. Patients with documented cardiovascular or cerebrovascular disease or malignancy were also excluded.

Control subjects were recruited from the cardiology clinic among individuals who presented with nonspecific complaints including dyspepsia and noncardiac chest pain with incidental abdominal imaging available in the electronic medical records of the hospital. Subjects without cardiovascular disease, hypertension, diabetes mellitus, dyslipidaemia, obesity, and medication use were included as control subjects. Echocardiographic examination of both groups was performed by the same cardiologist. Ethics approval of the study was obtained from the university ethics committee. Helsinki Declarations were followed and written informed consent was obtained from all participants.

### 2.2. Cardiac assessment

Participants were assessed with Philips EPIQ 7 ultrasonography system and an S5–1 probe. Transthoracic M-mode, two-dimensional, and subsequent standard and pulsed tissue Doppler echocardiographic examinations were done at the lateral decubitus position. Images were reviewed by the same cardiologist blinded to the patients’ information. Left ventricular (LV) ejection fraction and the LV and left atrial diameters were measured on M-mode traces recorded in the parasternal long-axis view compatible with standards. LVM was calculated with Penn equation [12].

EATT was defined as the relatively echo-free space between the outer wall of the myocardium and the visceral layer of the pericardium. It was measured perpendicularly on the free wall of the right ventricle at end-systole in 3 cardiac cycles and measured perpendicularly from the free wall of the right ventricle at end-diastole. The maximum EATT was measured from the point on the free wall of the right ventricle along the mid-line of the ultrasound beam perpendicular to the aortic annulus as a landmark [13].

CIMT was measured with T8, a general electric ultrasonography system, and a 6–13 MHz GE L6–12-RS linear array transducer. It was defined as the distance between lumen-intima and the media-adventitia of the carotid arterial wall on the ultrasound. Carotid artery measurements were obtained in the supine position. The right and left common carotid artery proximal to the carotid bulb was imaged in multiple longitudinal planes for the best resolution of the intima media thickness of the far wall. CIMT was measured by manually tracing the intima-media wall of the artery. Three points were measured on one scan and were synchronized with R-wave peaks on the electrocardiography to avoid errors resulting from variable arterial compliance. The mean CIMT was calculated from six measurements taken from two scans [14].

### 2.3. Statistical analysis

Statistical analysis was performed using the SPSS software version 16. Normality of the variables was tested using visual (histogram) and analytic methods (Kolmogorov–Smirnov/Shapiro–Wilk’s test) to determine whether they are normally distributed. Mann–Whitney U test was conducted to compare parameters among groups. Pearson’s and Spearman’s tests were used to measure correlations between variables. Multiple linear regression analyses using the stepwise method were performed to assess the independent variables affecting the dependent variable CIMT. All independent variables in the multiple linear regression were checked for multicollinearity. If the variance inflation factor (VIF) exceeded 3.0, the variable was considered to be collinear. All reported confidence interval (CI) values were calculated at the 95% level. An overall 5% type-I error level was used to infer statistical significance.

## 3. Results

One hundred and twenty patients diagnosed with AI were evaluated. A total of 81 participants (40 female, 49.4%) who met the inclusion criteria were included in the study. There were 41 patients (21 female, 52.5%) in the AI group and 40 patients in the control group (19 female, 46.3%). The demographic characteristics of the participants are shown in Table 1. There were no significant differences with respect to age, gender, BMI, or smoking habits (pack-years) between the two groups. The majority of the adenomas were on the left side (26; 59%). Two patients had bilateral adenomas.

Patients with AI had significantly higher high-density lipoprotein (HDL) cholesterol levels than controls (57.2±16.7 vs. 45.4±18.0, p = 0.03). Control subjects had higher triglyceride levels than patients (132.3±70.7 vs.176.1±78.0, p = 0.02). Diastolic blood pressure of the control group was higher than the AI group (**p <** 0.001). Patients with AI had statistically greater CIMT values (0.57±0.15 vs. 0.49± 0.13, p = 0.038). No differences were observed between the two groups regarding LVM or EATT (Table 2). While the LV ejection fraction was within the normal range in both groups, it was lower in AI patients than in controls (64.0±7.5 vs. 68.5±5.2, p = 0.008). Mitral E was lower and mitral E deceleration time was longer in AI patients than in controls (69.5±17.3 vs. 79.9±20.2, p = 0.02; 178.0±47.5 vs. 127.1±33.6, **p <** 0.001; respectively). There was no correlation between CIMT, adenoma size and serum cortisol (p = 0.2 and p = 0.6, respectively). There was a statistically significant correlation between age and CIMT (p = 0.016, r = 0.295).

Parameters affecting CIMT were then evaluated by multivariate analysis. Statistically significant parameters and parameters likely to affect the CIMT were included in the model. In this context, the relationship between CIMT and age, gender, BMI, HbA1c, adenoma size, creatinine, LDL-C, HDL-C, systolic blood pressure (SBP), diastolic blood pressure (DBP), cortisol level, and smoking were evaluated. HBA1c (p = 0.001) and age (p = 0.05) were independently associated with CIMT in regression analysis (Table 3).

## 4. Discussion

In this study, we found that CIMT was increased in patients with AI compared to healthy controls. However, no significant difference was observed between the two groups with respect to LVM or EATT.

It is plausible that cardiovascular risk is increased in patients with an adrenal adenoma and subclinical Cushing’s syndrome. Subtle production of cortisol hormone can lead to metabolic abnormalities even in the absence of clinically apparent diabetes or hypertension [15]. Metabolic disturbances may lead to cardiac changes such as increased LVM and EATT, which is the visceral fat tissue of the heart. It has been suggested that excess EATT may contribute to increased LVM as an end-organ damage [16]. Still, the causes of these cardiac complications are not clearly understood.

Interestingly, even nonfunctioning adenomas have been shown to cause metabolic disturbances such as insulin resistance. Increased levels of proinflammatory cytokines have been proposed as a possible explanation for the metabolic dysfunction in nonfunctioning adrenal adenomas [17,18].

We found CIMT to be significantly increased in patients with AI. Our finding is in line with Imga et al.’s study assessing cardiovascular indices in patients with AI [19]. It is a relatively easy and noninvasive method for showing early signs of atherosclerosis and is considered reliable in this population. Evran et al. have proposed that CIMT be used as a risk indicator for metabolic syndrome screening in AI patients [20].

Iacobellis et al. [16] have shown that EATT and LVM are greater in patients with AI. However, the study participants were relatively older compared to our cohort. An older age group could have been presented with full-blown cardiac manifestations of AI, including increased EATT and LVM.

High-density lipoprotein (HDL) cholesterol levels of AI patients were higher, and triglyceride levels lower than controls. Hence, patients with AI had a better lipid profile, which might have neutralized the negative effect of AI on cardiac adipocytes. In addition, the control group had a higher diastolic blood pressure. The atherogenic lipid profile, together with higher diastolic blood pressure, might explain why we failed to show a significant difference in EATT and LVM between the two groups. Iacobellis et al. also reported higher HDL levels [16] and Imga et al. reported lower triglyceride levels in AI patients [19]. These incompatible results raise the question of whether there is a different mechanism responsible for the cardiac changes in AI. Ermetici et al. have investigated the effect of adipokines on AI and found higher levels of plasma interleukin-6 (IL-6), adiponectin, resistin, tumour necrosis factor alpha, and monocyte chemoattractant protein -1 in patients with AI compared to controls [17]. AI patients had significantly elevated IL-6, secreted from the adrenal gland, irrespective of plasma cortisol levels. Ermetici et al. have associated elevated adipokine levels with atherosclerosis and left ventricular remodelling regardless of the presence of obesity or insulin resistance [17]. In the present study, BMI values of AI patients were numerically greater than controls, although not statistically significant. It is possible that blood adipokine levels (rather than visceral fat, dyslipidaemia or insulin resistance) determine the extent of the cardiac damage in AI patients, which might explain why some patients undergo LVM and EATT changes and some do not. Our study would have been more conclusive if we had measured adipokine levels and correlated them with cardiac parameters.

While the LV ejection fraction was within the normal range in both groups, it was lower in AI patients than in controls. A subclinical impairment in LV systolic function may account for this finding. Subclinical impairment in LV systolic function might have been confirmed by reduced longitudinal strain in speckle tracking echocardiography in the AI group. Mitral E max values were lower, and deceleration time was longer in AI patients compared to healthy controls, indicating a diastolic dysfunction. Imga et al. has found no difference between patients and controls regarding EF [19]. De et al. have similarly reported reduced EF and E/A ratio (the ratio between E-wave and A-wave) in AI patients [21]. Even if the LV ejection fraction is preserved, it may be reasonable to monitor AI patients for both systolic and diastolic cardiac dysfunction.

HbA1c and age were the only parameters independently associated with CIMT in regression analysis. It is not unexpected that unregulated blood glucose and older age increase CIMT. However, a larger sample size might reveal further associations between CIMT and other metabolic parameters.

Our study has some limitations. First, our sample size was relatively small. Second, we were not able to relate the increased CIMT to increased cardiovascular events in AI patients; the clinical relevance of our findings remains to be elucidated. The fact that our study has different results compared to previous studies on AI patients indicate heterogeneity among AI patients. Further studies with larger sample sizes are warranted to explain the contradictory results in the literature.

Our study also has several strengths. Previous research has shown an increased occurrence of cardiovascular events related to the subclinical secretion of cortisol in AI patients [22]. However, research on the cardiovascular effects of nonfunctioning adrenal adenomas is still scarce. In addition, reports of AI generally have a higher ratio of female patients, whereas our study had a gender ratio of one.

In conclusion, we found that patients with AI had increased CIMT compared to controls. However, this difference was not observed for EATT or LVM. Cardiovascular risk is increased even in nonfunctioning adrenal incidentalomas; hence, patients need to be monitored for cardiac dysfunction. In light of our findings, CIMT may be used to assess early cardiovascular risk in relatively young AI patients. With the increased utilization of thoracic imaging due to the COVID-19 pandemic, clinicians will most likely see a surge in AI cases. Prospective studies with larger sample sizes should be designed to investigate the changes in CIMT, EATT, and LVM in AI patients and their relation to cardiovascular morbidity and mortality.

## Figures and Tables

**Table 1:** Demographic, laboratory, and clinical characteristics.

	Incidentaloma group (n = 41)	Control group (n = 40)	p value
**Age** (years)	52.9 ± 11.5	48.5 ± 10.9	0.08
**Male/female** (n/n)	19/22	21/19	0.65
**Body-mass index** (kg/m^2^)	25.5 ± 2.0	22.5 ± 2.1	0.23
**Systolic BP** (mmHg)	117.2 ± 8.9	117.5 ± 8.1	0.87
**Diastolic BP** (mmHg) median (IQR)*	70 (70–70)	80 (70–80)	**<0.00** ^1^ **
**Heart rate** (beat/min) median (IQR)*	70 (64–76)	75 (69,5–78)	0.057
**Triglycerid**e (mg/dL)	132.3 ± 70.7	176.1 ± 78.0	**0.02** **
**HDL cholestero**l (mg/dL)	57.2 ± 16.7	45.4 ± 18.0	**0.0** ^3^ **
**LDL cholesterol** (mg/dL)	143.2 ± 44.7	136.0 ± 36.7	0.49

* Mann–Whitney U test.

** Statistically significant.

BP: Blood pressure; HDL: High-density lipoprotein; IQR: Interquartile range; LDL: Low-density lipoprotein.

If not otherwise stated, all data are presented as mean ± standard deviation.

**Table 2:** Comparison of echocardiographic measurements.

	Incidentaloma group (n = 41)	Control group (n = 40)	p value
**IVS** (cm), median (IQR)*	0.9 (0.9–1,0)	1.0 (0.9–1.0)	0.43
**PW** (cm), median (IQR)*	0.9 (0.8–0.9)	0.9 (0.8–0.9)	0.62
**LVDD** (cm)	4.5 ± 0.3	4.6 ± 0.4	0.24
**LVSD** (cm)	2.6 ± 0.3	2.8 ± 0.3	**0.02** **
**EF** (%)	64.0 ± 7.5	68.5 ± 5.2	**0.008** **
**LVM** (cm)	158.3 ± 38.0	159.7 ± 39.3	0.86
**Mitral E max (cm/s)**	69.5 ± 17.3	79.9 ± 20.2	**0.02** **
**Mitral A max** (cm/s)	69.0 ± 15.5	65.5 ± 16.5	0.37
**Mitral E deceleration time** (ms)	178.0 ± 47.5	127.1 ± 33.6	**<0.001** **
**E’** (septal E)	7.8 ± 2.3	9.0 ± 2.4	0.4
**A’** (septal A)	8.5 ± 1.8	6.7 ± 2.1	**<0.001** **
**EATT** (mm)	4.8 ± 1.4	4.7 ± 0.9	0.82
**CIMT** (mm)	0.57 ± 0.15	0.49 ± 0.13	**0.038** **

* Mann–Whitney U test.

** Statistically significant.

IVS: Interventricular septum; PW: Posterior wall; LVDD: Left ventricular diastolic diameter; LVSD: Left ventricular systolic diameter; EF: Ejection fraction; LVM: Left ventricular mass; IQR: Interquartile range; EATT: Epicardial adipose tissue thickness; CIMT: Carotid intima-media thickness.

If not otherwise stated, all data is presented as mean ± standard deviation.

**Table 3:** Factors relaed to CIMT in stepwise multiple linear regression analysis.

Coefficients^a^				
Model	Unstandardized Coefficients		Standardized Coefficients	p value
	B	Std. Error	Beta	
(Constant)	−1.252	0.116		0.002
**HbA1c**	0.332	0.024	1.113	**0.001**
**Age**	−0.004	0.001	−0.258	**0.050**

Excluded Variables^a^				
Model	B	Partial Correlation	Collinearity Statistics	p value
			Tolerance	
**Male**	**−**0.082	**−**0.665	0.885	0.335
**BMI**	**−**0.106	**−**0.425	0.218	0.575
**Adenoma size**	**−**0.106	**−**0.577	0.405	0.423
**Creatinine**	**−**0.080	**−**0.510	0.551	0.490
**LDL cholesterol**	**−**0.009	0.096	**−**0.039	0.241
**HDL cholesterol**	0.067	0.412	0.511	0.588
**Systolic BP**	0.132	0.594	0.276	0.406
**Diastolic BP**	0.094	0.601	0.558	0.399
**Cortisol**	**−**0.077	**−**0.513	0.606	0.487
**Smoking**	**−**0.130	**−**0.406	0.133	0.594

a Dependent variable: CIMT.

b Correlates in the model: (Constant), HbA1c, age.

BMI: Body mass index; BP: Blood pressure; CIMT: Carotid intima-media thickness; HbA1c: Glycated hemoglobin; HDL: High-density lipoprotein; LDL: Low-density lipoprotein.
